# The Therapeutic Action of Minute and Frequently Repeated Doses of Medicines

**Published:** 1883-09-20

**Authors:** G. G. Roy

**Affiliations:** Professor of Materia Medica in Southern Medical College, Atlanta, Ga.


### Southern Medical Record:

EDITORS:

T. S. POWELL, M.D. W. T. GOLDSMITH, M.D. R. C. WORD, M.D.

*R. C. WORD, M.D., Managing Editor.*

☞ All Communications and Letters on Business connected with the RECORD must be addressed to the Managing Editor.

VOL. XIII. ATLANTA, GA., SEPTEMBER 20, 1883. NO. 9.

### ORIGINAL AND SELECTED ARTICLES.

### THE THERAPEUTIC ACTION OF MINUTE AND FREQUENTLY REPEATED DOSES OF MEDICINES.

BY G. G. ROY, M. D.,

Professor of Materia Medica in Southern Medical College.

About four years ago, a young lady from one of the lower counties of Georgia, was placed under my care for the treatment of a long continued uterine trouble with obstinate amenorrhœa.

After an operation upon the cervical canal—which relieved what was apparently the great trouble in her case—the obstinate amenorrhœa continued, with great irritability of the stomach in the face of the usual remedies administered in the ordinary doses for this sometimes exceedingly troublesome disorder.

Acting upon the suggestion of Ringer and others, I put the patient upon one drop doses of the fluid extract of ergot every hour with instructions to take two doses every two hours should she forget or from any cause fail to take the medicine every hour. This she did for a week or ten days, with improvement more prompt and satisfactory than I had anticipated.

From such satisfactory results, with minute doses of ergot, and still following the suggestions of Sydney Ringer, Bartholow and other modern therapeutists, I continued to prescribe minute and often repeated doses of other medicines, with a similar satisfactory result.

In cases of engorged liver, with irritable stomach, and frequently in a condition of that organ amounting to severe catarrh, I used calomel in the doses of from 1-12 to 1-5 of a grain combined with a little bicarb. of soda and administered it every two to three hours, with the effect of arousing the secretions and relieving the engorged cappillary condition of the organs involved, more promptly, more effectually and with less distress to the patient than it has been my good fortune to obtain with the ordinary larger doses administered in the usual way.

I have, too, succeeded in relieving bilious headache resulting from obstinate constipation with Warner & Co.'s granules of minute proportions of aloin, strychnia and belladonna administered several times during the day for a week or ten days. And long anterior to the knowledge of the existence ot these preparations of Warner's, I have succeeded effectually in relieving obstinate constipation with the extract of belladonna alone, administered in doses of 1-20 to 1-10 of a grain three times a day, and perseveringly continued until the desired result was manifest.

It is well to say, just here, that in some cases of obstinate habitual constipation our patience will become quite threadbare, and tired of waiting to see the desired effect of this remedy in these cases, but we must cling to the latin maxim of 'Perseverentia" etc., and my word for it your labor will not have been in vain, and you will live to see the day when your patients will begin to feel and look a little threadbare in flesh if the remedy is continuously persisted in.

I have also used this remedy—extract of belladonna—in similar small doses, but more frequently repeated, in facial and other forms of neuralgia, with marked relief to my patients.

In truth, I believe that the therapeutic value of belladonna as a remedy in many of the neuroses, and in those conditions in which the muscular fibrillæ of the intestines and other viscera of the abdominal and thoracic cavities have borne as great a tension or strain as it is possible for them to bear without a tranquilizing and tonic relief, is but poorly appreciated. Much of the prejudice against this medicine, which has, for a long time, existed with the regular profession, has been due to the extraordinary and miraculously vaunted virtues of the drug by the homeopathist and other rregular practitioners. Nothing so quickly injures a good cause as to be championed by disreputable advocates.

The resin of podophyllin is another remedy which has been given in minute doses, say, 1-10, 1-20 and 1-40 of a grain with great satisfaction, for the relief of obstinate constipation, torpid liver and all the unpleasant train of dyspeptic symptoms resulting from these conditions.

In doses of 1-40 of a grain night and morning—not sufficient to produce any purgation—podophyllin has been highly recommended for what has been termed its alterative effect; and a recent writer in the Chicago Medical Times, says it is highly useful in a case like this, *i.e.*, "A busy, worried, overworked man, who takes perhaps too little exercise—feels all day, but especially in the morning—dull, depressed; his mind inactive and indolent, and he is irritable.

He has, perhaps, a stupid feeling; he is often bilious-looking, and is dark around the eyes. Now, these symptoms, no doubt, often accompany sluggish bowels, and can be relieved by any purgative, but they not uncommonly occur where the bowels are regular and the motions natural in color. *In all such cases* the small non-purgative doses of podophyllin are most serviceable."

Aconite is another remedy whose administration in minute doses has given excellent results, "Especially for the purpose of reducing temperature and checking inflammatory process." This remedy receives the highest recommendation from both Ringer and Bartholow. The latter speaks of it as a powerful agent which will produce manifest results in small doses.

Nux Vomica, according to Ringer, is possessed of real curative powers for sick-headache accompanied with acute gastric catarrh, whether due to error in diet, constipation or no apparent cause. He also regards it, administered in small and frequently repeated doses, as useful in many disturbances of the gastric function."

Dr. A. A. Smith, Prof. of Materia Medica and Therapeutics and of Clinical Medicine in Bellevue Medical College, in a recent lecture, says: "One of the most important remedies which can be administered with great benefit in frequently repeated doses, is ipecac." "We have all been educated to believe—as taught also by experience—that this medicine is a most certain and efficient emetic; but experience has likewise demonstrated the fact that a single dose of the wine of ipecac will often arrest obstinate vomiting. It should by repeated every 10 or 15 minutes. When administered in this manner he has often known it to relieve vomiting from different causes, among which are pregnancy and subacute gastritis. Children often vomit from very slight causes, and are likely to suffer from diarrhœa and vomiting which has no other assignable cause than disturbance of digestion."

A single drop of the wine of ipecac, repeated every 15 or 20 minutes, will often produce the most marked relief, both from the vomiting and diarrhœa.

Thus administered the drug is not nauseating and is easily taken.

Upon the authority of Trouseau and his successor, he asserts what at one time appeared to him to be incredible, viz: that one sixtieth of a grain of calomel taken every hour for ten or twelve hours will relieve the headache of syphilis occurring at night. Dr. Smith says that he has given 1-40th of a grain doses in this manner and obtained the results which they claim for it, but he has not tried it in doses of 1-60th of a grain. He found marked relief by the second or third night.

"One-twenty-fourth of a grain of mercury with chalk, administered every 15 or 20 minutes is often of great benefit in the vomiting, and non-inflammatory diarrhœa of children."

A single drop of the tincture of nux vomica, given every 10 minutes, will often produce most marked relief in sick-headache not of neurotic origin. It should be given immediately or soon after meals.

I am sure you will find, in organic diseases of the heart, where digetalis is indicated—if given in minute doses, say one drop of the tincture every hour or half-hour, its beneficial effects are more certain and satisfactory than if administered as it is ordinarily, in 10 or 15 drop doses three times a day.

In neuralgias of the face and head, the strong tincture of gelseminum, if given in one drop doses every hour or half hour are much more efficacious than administered in the ordinary doses at the usual intervals.

I must refer you to Ringer's "Handbook of Therapeutics" for his experience in the use of minute doses of tartar emetic in bronchitis of children—of minute doses of mercury in cases of syphilitic headache, (already referred to) and corrosive sublimate in a certain variety of the diarrhœa of children—of this latter he says:

"In a form of diarrhœa in children likely to be mistaken for dysentery, but when the general symptoms are mild, and the special features are secondary to the diarrhœa, corrosive sublimate will be found to render most satisfactory service in effecting a cure." The principal indication for the use of corrosive sublimate, he says, "is the mucous character of the stools, whether containing blood or not."

Time will not permit me to further pursue this list of remedies whose efficiency has been tested by the unfailing rule of experiment and experience, but the profession has been awakened to the advantages of this matter by the writings of Ringer, Bartholow and others, and I think we can anticipate the day when this mode of administration of remedies will be given a thorough trial by the members of the regular medical profession.

If you ask me the reason for the enhanced efficacy of remedies thus administered, I cannot tell you Bartholow says that the therapeutical action is the physiological antagonist of the diseased action.

"The only certain principle of action, however, is found in actual experience."

Should this mode of administration stand the test of thorough experience, physicians will have found a long-desired boon in the treatment of children, which is often so unsatisfactory in consequence of a natural repugnance to all medicines.

